# Comparison of the frequency of periodontal pathogenic species of diabetics and non‐diabetics and its relation to periodontitis severity, glycemic control and body mass index

**DOI:** 10.1002/cre2.453

**Published:** 2021-05-26

**Authors:** Mojtaba Akherati, Ebrahim Shafaei, Hamid Salehiniya, Hamid Abbaszadeh

**Affiliations:** ^1^ Student Research Committee Birjand University of Medical Sciences Birjand Iran; ^2^ Infectious Diseases Research Center Birjand University of Medical Sciences Birjand Iran; ^3^ Social Determinants of Health Research Center Birjand University of Medical Sciences Birjand Iran; ^4^ Department of Oral and Maxillofacial Pathology Faculty of Dentistry, Birjand University of Medical Sciences Birjand Iran

**Keywords:** bacteria, body mass index, diabetes mellitus, periodontal disease, periodontal index

## Abstract

**Objectives:**

To investigate common pathogenic bacteria of periodontal diseases (PD) in patients with type 2 diabetes mellitus (T2DM) and its relationship with PD severity, glycemic control and body mass index (BMI).

**Material and Methods:**

This case–control study consisted of 55 patients with T2DM and 55 individuals as control. Samples were collected from periodontal pockets. After DNA extraction, using 16srRNA‐specific primers, the presence of *Aggregatibacter actinomycetemcomitans* (Aa), *Porphyromonas gingivalis* (Pg), *Tannerella forsythia* (Tf), *Prevotella intermedia* (Pi), and *Fusobacterium nucleatum* (Fn) were examined based on polymerase chain reaction method.

**Results:**

Aa frequency was significantly higher in in T2DM group than control. There were no significant differences in the frequencies of Pg, Tf, Pi, and Fn between studied groups. There were no significant differences between frequencies of studied bacteria in different severities of periodontitis in T2DM group. Prevalence of Tf in T2DM patients with moderate periodontitis was significantly higher than non‐diabetics with moderate periodontitis. There was no significant difference between the frequency of bacteria in diabetics with good and poor glycemic control. There was a significant difference between the frequencies of Pg in T2DM individuals with different BMI levels.

**Conclusions:**

A higher frequency of detection of Aa was found in diabetic when compared to non‐diabetics. Glycemic control did not affect the frequency distribution of studied bacteria in T2DM. Pg was identified in higher frequency in overweight T2DM patients.

## 1 INTRODUCTION

Periodontal disease (PD) is an inflammatory and infectious disease of the supporting tissues around the tooth, which originates from microbial plaque‐derived product and occurs due to a specific and complex interaction between pathogenic bacteria and the host response (D'Cruz, 2020).

Diabetes mellitus (DM) refers to a group of metabolic diseases that result from any defect in insulin secretion, insulin action, or both. Type 2 diabetes mellitus (T2DM) often develops as a result of insulin resistance along with a gradual loss of insulin secretion by beta cells of islets of Langerhans (American Diabetes Association, 2020).

Periodontitis has been suggested to be often associated with diabetes and may be a chronic complication of DM, in both type 1 and type 2 (Tam et al., 2018).

PD is more common along with greater severity in diabetics. The greater susceptibility is not associated with higher plaque and calculus. Existence of certain subgingival microbial organisms may be a risk factor. PD management in diabetics is associated with challenges and may need to modify the treatment plan (Campus et al., 2005).

Changes in host response and sub‐gingival microbial pathogens are suggested determinants for higher susceptibility of diabetics to PD. Only a few studies have focused on the effect of glycemic control on the sub‐gingival microbial pathogens (Miranda et al., 2017).

T2DM and obesity are associated with periodontal inflammation. The pro inflammatory environment associated with both obesity and T2DM may link these two diseases with PD. Alteration in periodontal pathogenic bacteria as a possible linkage between PD, T2DM and obesity is still not well recognized (Tam et al., 2018).

The relationship between diabetes and PD is bilateral in that inflammatory mediators may adversely affect glucose control, and elevated glucose levels and glycation end products may alter the host response to bacterial infections. However, no agreement has been reached on the effect of DM on the subgingival microbiota (Chakraborty & Ghosh, 2019). In more studies, the findings indicate that there are differences in the subgingival microbiota between diabetic and non‐diabetic patients (Aemaimanan et al., 2013; Castrillon et al., 2015; Ebersole et al., 2008; Sardi et al., 2011; Shi et al., 2020; Zhou et al., 2013). On the other hand, in the mentioned studies, there is a difference of opinion between the types of bacteria increased in the subgingival microbiota in diabetic patients. For example, in Castrillon et al. study (Castrillon et al., 2015), red complex microorganisms were detected in lower frequencies in patients with diabetes but in Aemaimanan et al. study (Aemaimanan et al., 2013) the high quantity of red complex bacteria was detected in DM patients. In contrast, other studies show that type 2 diabetes has no significant influence on the prevalence of the periodontal pathogens (Hintao et al., 2007; Joaquim et al., 2018; Mohamed et al., 2016; Yuan et al., 2001).

So, the primary aim of this study was to compare the frequency of five pathogenic species in the subgingival microbiota of diabetics and non‐diabetics. Secondary aim was to evaluate the frequency of pathogens in diabetic patients in relation to periodontitis severity, glycemic control and body mass index (BMI).

## 2 MATERIALS AND METHODS

This study was approved by ethic committee of the University. Study participants provided informed consent.

### 2.1 Selection of the study population

In this case–control study, the study population included 55 patients with T2DM who were selected from the patients referred to the diabetes clinic of Valiasr Hospital of Birjand University of Medical Sciences. The control group also included 55 healthy non‐diabetic individuals who were selected from the patients referred to the clinic of Birjand Dental School. Informed consent was obtained from the individuals.

Diagnosis of DM was made according to criteria defined by the American Diabetes Association (ADA) and included: “Fasting plasma glucose (FPG) level of 126 mg/dl (7 mmol/L) or higher” or “Two‐hour plasma glucose 200 mg/L (11.1 mmol/L) or higher during a 75 g oral glucose tolerance test (OGTT)”or“ random plasma glucose 200 mg/dl or higher in a patient with classic symptoms of hyperglycemia or hyperglycemic crisis” or “The hemoglobin A1c (HbA1c) level 6.5% or higher”. Confirmation of the diagnosis of DM required two abnormal test results from a single sample or two separate test samples (American Diabetes Association, 2020). For a patient to be included in T2DM group in our study, 1 year had passed since the initial diagnosis of diabetes (Tam et al., 2018). Exclusion criteria were: history of infectious disease in a recent year, history of immunosuppressive drugs, presence of less than 6 teeth in the mouth, having type 1 diabetes mellitus, chemotherapy and history of radiotherapy, smoking more than five cigarettes a week, alcohol consumption more than 40 grams of alcohol per day, use of drugs that affect the condition of the gingiva (e.g., phenytoin), prophylaxis for endocarditis (Tam et al., 2018), presence of aggressive periodontitis, pregnancy, breastfeeding, history of other systemic diseases (e.g., cardiovascular), treatment with antibiotics 3 months before the start of the study, long‐term use of anti‐inflammatory drugs, receive periodontal treatments within the last 6 months (Casarin et al., 2013). For persons to be included as control group HbA1c test was performed and HbA1c should be below 5.7%; Individuals who their HbA1c was between 5.7% and 6.4% was considered prediabetes and excluded from control group (Casarin et al., 2013).

Patients' glycemic control was based on HbA1c levels and were categorized into good control <7%, and poor control ≥7% (American Diabetes Association, 2020).

Based on the presence or absence of periodontitis, the diabetic and control groups were divided into two subgroups: with chronic periodontitis and without chronic periodontitis. To be included in the definition of chronic periodontitis, the amount of bone destruction and attachment loss had to be consistent with the presence of local factors, such as biofilm, calculus etc. and this destruction occurred mainly with mild to moderate progression rate. Also, periodontitis group were classified into three subcategories according to severity of periodontitis: mild, moderate and severe. Criteria for diagnosis of periodontitis according to its severity level (mild, moderate, severe and no periodontitis) were based on Gomes‐Filho et al., modified (Gomes‐Filho et al., 2018) (The difference between the Gomes‐Filho et al. criteria and Gomes‐Filho et al., modified criteria is only the minimum number of teeth considered: at least 2 teeth for the criterion Gomes‐Filho et al., modified and at least 4 teeth for Gomes‐Filho et al.).

### 2.2 Measurement of periodontal parameters

Periodontal parameters including presence of calculus, plaque index (PI), bleeding on probing (BOP), pocket depth (PD), clinical attachment level (CAL) and community periodontal index (CPI) index were assessed. Periodontal parameters were assessed at four sites per tooth (mesiofacial papilla, distofacial papilla, facial margin and lingual margin) excluding third molars using the WHO periodontal probe.

PD was calculated by measuring the distance from the base of the pocket or gingival sulcus to the gingival margin. PD was measured for each tooth at the above‐mentioned four areas (mesiofacial papilla, distofacial papilla, facial margin and lingual margin) and the average of these 4 numbers was recorded as the PD for that tooth. If any of the teeth were missing, that tooth was removed from the calculations. Third molars were not included in this study. Pockets with a depth of 1 to 3 mm were considered normal and pockets with a depth of ≥4 mm were defined as periodontal pocket; the percentage of pockets ≥4 mm was calculated and recorded for each patient (Campus et al., 2005).

CAL was calculated by measuring the distance from the base of the pocket or gingival sulcus to the cement‐enamel junction (CEJ). Numbers of sites with CAL less than 3, 3–4, ≥5 mm were recorded (Alasqah et al., 2018).

BOP determination was based on stimulation of the gingival tissue using a probe in which the probe is inserted into the base of the gingival sulcus/pocket with a standard force (20 g probing force). To determine the BOP score, the percentage of BOP‐positive sites was calculated (Miranda et al., 2017).

To determine the PI, Silness‐Löe PI was used, where the PI is determined based on soft debris on all teeth and a score is given from 0 to 3 (0 = No plaque;1 = A film of plaque adhering to the free gingival margin and adjacent area of the tooth. The plaque may be seen in situ only after application of disclosing solution or by using the probe on the tooth surface; 2 = Moderate accumulation of soft deposits within the gingival pocket, or the tooth and gingival margin which can be seen with the naked eye; 3 = Abundance of soft matter within the gingival pocket and/or on the tooth and gingival margin. The indices for the following six teeth (index teeth) were considered to designate the PI: 16, 12, 24, 36, 32, and 44.The index for the patient is obtained by summing the indices for all six teeth and dividing by six (Tam et al., 2018).

To determine the CPI index, the mouth was divided into sextants defined by tooth numbers: 18–14, 13–23, 24–28, 38–34, 33–43, and 44–48. A sextant was examined only if there are two or more teeth present. Since the subjects were over 20 years old, 10 index teeth were examined, each of which represents a sextant (teeth 17/16, 11, 26/27, 47/46, 31, 36/37). The two molars in each posterior sextant were paired for recording, and if one was missing, there was no replacement. If no index teeth or tooth was present in a sextant qualifying for examination, all the remaining teeth in that sextant were examined and the highest score is recorded as the score for the sextant. After examination of four sites (labial, lingual/palatal, mesial, and distal) in index teeth, the highest CPI score in each sextant was considered as that sextant score. The index teeth were probed and the highest score recorded in the appropriate box. The codes were: 0 = Healthy; 1 = Bleeding observed, directly or by using mouth mirror, after probing; 2 = Calculus detected during probing, but all the black band on the probe visible; 3 = Pocket 4–5 mm (gingival margin within the black band on the probe); 4 = Pocket 6 mm or more (black band on the probe not visible); X = Excluded sextant (less than two teeth present). After examining and determining the sextant scores, the number of sextants related to each code was recorded. To compare between healthy and diabetic individuals, the number of sextants related to each code was compared between the two groups (Kim et al., 2013).

### 2.3 Sampling of subgingival microbiota

To collect samples, samples of individuals without periodontitis were collected from mesiobuccal and mesiolingual sites of the first maxillary and mandibular molars (8 sites in each people) (Jardim Júnior et al., 2006). The samples from the 8 sites were pooled in the same tube and afterwards one final sample was evaluated per subject (PCR of one pooled sample per patient). The first molar teeth showing furcation lesions, endodontic pathology, or extensive crown destruction were excluded from sampling. In the absence of one or more of the above teeth, the shortage of sample collection sites was compensated. Non‐adjacent gingival sulcus was randomly selected as follows. For all sites (gingival sulcus) a paper code was created and placed in an opaque envelope. Subsequently, by drawing paper (lottery), a number of sites appropriate to the shortage of sampling sites were selected for sampling and analysis of sub‐gingival biofilm (Casarin et al., 2013). In patients with periodontitis, like individuals without periodontitis, samples were collected from deepest area of mesiobuccal and mesiolingual sites of the first maxillary and mandibular molars (8 sites in each people) (Jardim Júnior et al., 2006).

After careful removal of the supragingival biofilm using a sterile swab, the area was washed with water spray, isolated with a cotton roll and gently dried. A sterile paper cone (No. 35, Meta Company, South Korea) was inserted into the bottom (base) of the sulcus/pocket for 30 s and the paper cone was placed in sterile tube containing 200 μl of lysis fluid (Tris‐EDTA ‐ triton X100, PH 7.8).

### 2.4 DNA extraction and amplification

DNA extraction of the samples was performed by boiling method (for 10 min) after transfer to the laboratory. Then, using the specific 16S rRNA primers described in Table 1, the presence of subgingival microbiota for each of the bacteria *Aggregatibacter actinomycetemcomitans* (Aa), *Porphyromonas gingivalis* (Pg), *Tannerella forsythia* (Tf), *Prevotella intermedia* (Pi), and *Fusobacterium nucleatum* (Fn) was examined by Polymerase chain reaction (PCR).

**TABLE 1:** Demographic data of study participants

Group	Type 2 diabetes N (%)/mean ± SD	Control N (%)/mean ± SD	*p*‐value
Variable			
**Gender**			
**Male**	26 (47.3%)	23 (41.8%)	0.56
**Female**	29 (52.7%)	32 (58.2%)	
**Age** (years)	48.6 ± 8.9	46.1 ± 8.5	0.12
**Education level**			
**Illiterate or primary**	13 (23.6%)	10 (18.2%)	0.15
**Secondary or high school**	24 (43.6%)	17 (30.9%)	
**University**	18 (32.7%)	28 (50.9%)	
**Body mass index** (kg/m^2^)	28.3 ± 2.8	26.3 ± 3.3	0.001
**HbA1c level**	8.07 ± 1.32	5.04 ± 0.52	<0.001

For gene amplification, after providing the PCR condition and reaction mixture and using the thermal cycle mentioned in Table 1, amplification was performed for each bacterium in the thermocycler (Master cycler X50, Eppendorf Company, Germany).

### 2.5 Statistical analysis

The collected data were entered into SPSS software version 22. In order to analyze the data, Chi‐square test (for comparison of gender, level of education, frequency distribution of five pathogenic bacteria in subgingival microbiota between T2DM and non‐diabetics groups, frequency distribution of five pathogenic bacteria in relation to severity of periodontitis in T2DM and non‐diabetics groups, frequency distribution of five pathogenic bacteria in similar severity of periodontitis between T2DM and non‐diabetics groups, frequency distribution of five pathogenic bacteria in relation to glycemic control in T2DM group, and frequency distribution of five pathogenic bacteria in relation to BMI in T2DM and non‐diabetics groups), Fishers exact test (for comparison of frequency distribution of five pathogenic bacteria in similar severity of periodontitis between T2DM and non‐diabetics groups), and independent t‐test (for comparison of age, BMI, missing teeth, presence of calculus, BOP, PI, PD, CAL and CPI between T2DM and non‐diabetics groups) were used according to the type of data distribution at the significance level of 0.05.

## 3 RESULTS

Table 1 shows demographic data of 110 study participants (Table 1). There were not significant differences between diabetic and non‐diabetics groups according to gender, age, and education level (*p*‐value > 0.05). There was significant difference between these two groups according to BMI and HbA1c level (*p*‐value < 0.05).

Table 2 shows frequency distribution of study participants according to severity of periodontitis (Table 2).

**TABLE 2:** Frequency distribution of study participants according to severity of periodontitis

Group	Type 2 diabetes *N* (%)	Control *N* (%)
Variable		
**Severity of periodontitis**		
**No periodontitis**	5 (9.1%)	15 (27.3%)
**Mild**	9 (16.4%)	19 (34.5%)
**Moderate**	22 (40%)	14 (25.5%)
**Severe**	19 (34.5%)	7 (12.7%)

Based on the results of the present study, the mean number of missing teeth, presence of calculus, BOP, PI, PD, CAL, and CPI in the T2DM group were significantly higher than the C group (*p* < 0.05) (Table 3).

**TABLE 3:** Comparison of periodontal parameters in studied groups

Group	Diabetes mellitus	Control	*p*‐value
Parameter			
Missing teeth	4.4 ± 2.5	3.4 ± 2.3	0.035
Calculus	40.1 ± 11.9	31.0 ± 12.8	<0.001
Bleeding on probing	49.6 ± 11.9	34.1 ± 11.8	<0.001
Plaque index	1.8 ± 0.4	1.5 ± 0.4	<0.001
Pocket depth	9.4 ± 2.4	5.2 ± 2.3	<0.001
Percentage of Pocket ≥4 mm	72.7%	90.9%	<0.001
Community periodontal index	0.67 ± 0.77	0.38 ± 0.59	0.029
Clinical attachment loss	4.7 ± 1.3	2.5 ± 2.1	<0.001

Based on the results of the present study, there were no significant differences in the frequency distribution of Fn, Pi, Tf, and Pg in the two studied groups (*p* > 0.05). There was a significant difference between the frequency distribution of Aa in T2DM and C groups (*p* < 0.05), which was significantly higher in T2DM group (Table 4).

**TABLE 4:** Frequency distribution of bacterial species in studied groups

Group	Diabetes mellitus *n* (%)		Control *n* (%)		*p*‐value
Bacteria	Positive	Negative	Positive	Negative	
*Fusobacterium nucleatum*	25 (45.5)	30 (54.5)	16 (29.1)	39 (70.9)	0.076
*Prevotella intermedia*	35 (63.6)	20 (36.4)	32 (58.2)	23 (41.8)	0.558
*Tannerella forsythia*	16 (29.1)	39 (70.9)	12 (21.8)	43 (78.2)	0.381
*Porphyromonas gingivalis*	18 (32.7)	37 (67.3)	23 (41.8)	32 (58.2)	0.324
*Aggregatibacter actinomycetemcomitans*	11 (20)	44 (80)	3 (5.5)	52 (94.5)	0.022

Table 5 shows comparison of frequency distribution of bacterial species between T2DM group and non‐diabetics in similar severity of periodontitis (Table 5). Except for the level of Tf, which in moderate periodontitis was significantly different between diabetic and non‐diabetic groups (higher frequency in T2DM group), in other cases, the levels of studied bacteria at similar severity of periodontitis did not show significant differences between diabetic and non‐diabetic groups.

**TABLE 5:** Comparison of frequency distribution of bacterial species between T2DM group and non‐diabetics in similar severity of periodontitis

Group	Diabetes mellitus *n* (%)		Control *n* (%)		*p*‐value
Bacteria	Positive	Negative	Positive	Negative	
*Fusobacterium nucleatum*					
No periodontitis	1 (20)	4 (80)	2 (13.3)	13 (86.7)	0.06
Mild	5 (55.6)	4 (44.4)	5 (26.3)	14 (73.7)	0.21
Moderate	12 (54.5)	10 (45.5)	4 (28.6)	10 (71.4)	0.17
Severe	7 (36.8)	12 (63.2)	5 (71.4)	2 (28.6)	0.19
*Prevotella intermedia*					
No periodontitis	3 (60)	2 (40)	5 (33.3)	10 (66.7)	0.60
Mild	7 (77.8)	2 (22.2)	14 (73.7)	5 (26.3)	0.82
Moderate	16 (72.7)	6 (27.3)	10 (71.4)	4 (28.6)	0.93
Severe	9 (47.4)	10 (52.6)	3 (42.9)	4 (57.1)	0.84
*Tannerella forsythia*					
No periodontitis	2 (40)	3 (60)	6 (40)	9 (60)	1
Mild	3 (33.3)	6 (66.7)	4 (21.1)	15 (78.9)	0.65
Moderate	7 (31.8)	15 (68.2)	0 (0)	14 (100)	0.03
Severe	4 (21.1)	15 (78.9)	2 (28.6)	5 (71.4)	1
*Porphyromonas gingivalis*					
No periodontitis	1 (20)	4 (80)	8 (53.3)	7 (46.7)	0.31
Mild	2 (22.2)	7 (77.8)	6 (31.6)	13 (68.4)	0.69
Moderate	6 (27.3)	16 (72.7)	8 (57.1)	6 (42.9)	0.74
Severe	9 (47.4)	10 (52.6)	1 (14.3)	6 (85.7)	0.19
*Aggregatibacter actinomycetemcomitans*					
No periodontitis	0 (0)	5 (100)	0 (0)	15 (100)	‐
Mild	1 (11.1)	8 (88.9)	1 (5.3)	18 (94.7)	0.58
Moderate	5 (22.7)	17 (77.3)	0 (0)	14 (100)	0.13
Severe	5 (26.3)	14 (73.7)	2 (28.6)	5 (71.4)	1

There were no significant differences between the frequency distribution of Pi (*p* = 0.061), Tf (*p* = 0.072) and Pg (*p* = 0.160) in control individuals with different periodontitis severity (*p* > 0.05). There were significant differences between the frequency distribution of Fn (*p* = 0.047) and Aa (*p* = 0.030) in control individuals with different periodontitis severity (*p* < 0.05). Fn and Aa were present in higher frequencies in severe periodontitis than the other severity levels of periodontitis. There were no significant differences between the frequency distribution of Pi (*p* = 0.288), Tf (*p* = 0.791), Pg (*p* = 0.397), Fn (*p* = 0.395), and Aa (*p* = 0.518) in T2DM individuals with different periodontitis severity (*p* > 0.05). It means that frequency of each of studied bacteria in no periodontitis, mild, moderate and severe periodontitis subgroups were to some extent similar.

There were no significant differences between the frequency distribution of Fn, Pi, Tf, Pg, and Aa in T2DM individuals with different glycemic control (HbA1c level) (*p* > 0.05) (Table 6). Also, there were no significant differences between the frequency distribution of Fn (*p* = 0.843), Pi (*p* = 0.082), Tf (*p* = 0.871), Pg (*p* = 0.208), and Aa (*p* = 0.176) in T2DM individuals with different fasting blood sugar (FBS) level (<130 and ≥130 mg/dl) (*p* > 0.05).

**TABLE 6:** Frequency distribution of studied bacteria in T2DM individuals with different glycemic control

HbA1c level	<7	≥7	*p*‐value
Bacteria	*N* (%)	*N* (%)	
*Fusobacterium nucleatum*			
Negative	8 (50)	22 (56.4)	0.665
Positive	8 (50)	17 (43.6)	
*Prevotella intermedia*			
Negative	4 (25)	16 (41)	0.262
Positive	12 (75)	23 (59)	
*Tannerella forsythia*			
Negative	12 (75)	27 (69.2)	0.754
Positive	4 (25)	12 (30.8)	
*Porphyromonas gingivalis*			
Negative	12 (75)	25 (64.1)	0.434
Positive	4 (25)	14 (35.9)	
*Aggregatibacter actinomycetemcomitans*			
Negative	14 (87.5)	30 (76.9)	0.312
Positive	2 (12.5)	9 (23.1)	

There was no significant difference between the frequency distribution of Fn, Pi, Tf, Pg and Aa in the control individuals with different BMI levels (*p* > 0.05) (Table 7). There was no significant difference between the frequency distribution of Fn, Pi, Tf, and Aa in the T2DM individuals with different BMI levels (*p* > 0.05) (Table 7). There was significant difference between the frequency distribution of Pg in the T2DM individuals with different BMI levels (*p* < 0.05) (Table 7). Pg was identified in half of the overweight T2DM individuals, while it was not almost detected in normal BMI and obese groups.

**TABLE 7:** Frequency distribution of studied bacteria in the studied groups with different body mass index (BMI) levels

Group	BMI level	18.5–24.9	25–29.9	≥30	*p*‐value
	Bacteria	*N* (%)	*N* (%)	*N* (%)	
Control	*Fusobacterium nucleatum*				
	Negative	17 (81)	15 (60)	7 (77.8)	0.262
	Positive	4 (19)	10 (40)	2 (22.2)	
	*Prevotella intermedia*				
	Negative	1 (38.1)	10 (40)	5 (55.6)	0.653
	Positive	13 (61.9)	15 (60)	4 (44.4)	
	*Tannerella forsythia*				
	Negative	18 (87.5)	19 (76)	6 (66.7)	0480
	Positive	2 (14.3)	6 (24)	3 (33.3)	
	*Porphyromonas gingivalis*				
	Negative	9 (42.9)	17 (68)	6 (66.7)	0.194
	Positive	12 (57.1)	8 (32)	3 (33.3)	
	*Aggregatibacter actinomycetemcomitans*				
	Negative	21 (100)	22 (88)	9 (100)	0.149
	Positive	0 (0)	3 (12)	0 (0)	
Diabetics	*Fusobacterium nucleatum*				
	Negative	3 (42.9)	21 (61.8)	6 (42.9)	0.392
	Positive	4 (57.1)	13 (38.2)	8 (57.1)	
	*Prevotella intermedia*				
	Negative	2 (28.6)	13 (38.2)	5 (35.7)	0.238
	Positive	5 (71.4)	21 (61.8)	9 (64.3)	
	*Tannerella forsythia*				
	Negative	5 (71.4)	25 (73.5)	9 (64.3)	0.814
	Positive	2 (28.6)	9 (26.5)	5 (35.7)	
	*Porphyromonas gingivalis*				
	Negative	7 (100)	17 (50)	13 (92.9)	0.002
	Positive	0 (0)	17 (50)	1 (7.1)	
	*Aggregatibacter actinomycetemcomitans*				
	Negative	6 (85.7)	29 (85.3)	9 (64.3)	0.235
	Positive	1 (14.3)	5 (14.7)	5 (35.7)	

## 4 DISCUSSION

### 4.1 Oral microbiota, DM and PD

As mentioned, PD occurs due to the complex interaction between oral microbiota and the host response. In our study, Aa frequency was significantly higher in T2DM group. This finding may implicate that there is some relationships between the subgingival microbiota and DM; it is likely that DM can alter oral microbiota and accordingly can help to progression of PD.

There is no consensus on the effect of DM on subgingival pathogenic bacteria and the effect on the prevalence and level of these bacteria has not been properly elucidated. Some studies suggest that there are differences (in prevalence, level or both) in the subgingival microbiota between diabetics and non‐diabetics (Aemaimanan et al., 2013; Castrillon et al., 2015; Ebersole et al., 2008; Sardi et al., 2011; Shi et al., 2020; Zhou et al., 2013); other studies claimed that there is no difference in the subgingival microbiota between these two groups (Hintao et al., 2007; Joaquim et al., 2018; Mohamed et al., 2016; Yuan et al., 2001). The result of recent studies is in contrast to our findings because in our study the prevalence of Aa pathogen was significantly higher in DM patients than non‐DM patients. Since the literature suggests that the distribution and prevalence of periodontal pathogens vary depending on geographic locations as well as among different ethnic groups (Aemaimanan et al., 2013), this apparent discrepancy may be the result of the study populations derived from varied geographic locations as well as methodological differences. This discrepancy may also be because of differences among studies in the disease status of populations examined, site selection, sampling strategy, and the detection methods used.

In Castrillon et al. and Casarin et al. studies (Casarin et al., 2013; Castrillon et al., 2015), *P. gingivalis* (Pg), *Treponema denticola* (Td), and *T. forsythia* (Tf) were detected in lower frequencies in patients with DM. The detection of *A. actinomycetemcomitans* (Aa) was higher in diabetics compared to non‐diabetics. Their justification for the lower frequency of red complex bacteria (Pg, Td and Tf) in diabetic patients was that these bacteria are asaccharolytic and high glucose concentration in plasma and gingival crevicular fluid (GCF) of DM patients makes the environment unsuitable for the growth of these bacteria. In contrast, *A. actinomycetemcomitans* (Aa) has the ability to ferment different sugars, including glucose, and high glucose concentration in plasma and GCF of diabetics is in favor of the growth this bacteria.

In Aemaimanan et al. study (Aemaimanan et al., 2013), the high quantity of red complex bacteria was detected in DM patients than non‐diabetics. Since the bidirectional link between DM and PD has been extensively described, they hypothesized that co‐occurrence of Pg, Td and Tf, could play an important role in the alterations promoted by DM on periodontitis development. In fact, the consortium of these three bacteria is associated with chronic periodontitis and the synergistic virulence result in alveolar bone loss. On the other hand, in their study, the non‐diabetics were significantly younger than diabetics. So it was rumored that maybe higher quantities of periodontal pathogens in diabetic group were associated with subject age.

In Campus et al. study (Campus et al., 2005), prevalence of Tf was higher in the non‐diabetic group than in the T2DM group; this finding is consistent with the result of Castrillon et al. and Casarin et al. studies (Casarin et al., 2013; Castrillon et al., 2015), and is inconsistent with the result of Aemaimanan et al. study (Aemaimanan et al., 2013); in Campus et al. study (Campus et al., 2005) prevalence of Pg was higher in the T2DM group than in the non‐diabetic group; this finding is inconsistent with the result of Castrillon et al. and Casarin et al. studies (Casarin et al., 2013; Castrillon et al., 2015), and is consistent with the result of Aemaimanan et al. study (Aemaimanan et al., 2013). These inconsistencies can be attributed to the differences in sample size, glycemic control of the DM group, the average duration of disease (diabetes), distribution of periodontitis severity, and demographic characteristics of studied groups such as age, sex, race, etc.

### 4.2 BMI, DM and PD

In the present study, there was significant difference between the frequency distribution of Pg in the T2DM individuals with different BMI levels and Pg was identified in half of the overweight T2DM individuals. It can implicate that BMI level can affect the subgingival microbiota in T2DM patients.

In Tam et al. study (Tam et al., 2018), differences in composition and diversity of oral microbiota between obese and non‐obese patients with type 2 diabetes mellitus (T2DM) were statistically significant. Their conclusion about the effect of BMI on the subgingival microbiota in patients with T2DM is consistent with the result of our study.

### 4.3 Glycemic control, DM and PD

In Tam et al. study (Tam et al., 2018), cross‐sectional and longitudinal approaches failed to reveal statistically significant associations between HbA1c level and species composition or diversity. Their finding is consistent with our result.

In Aemaimanan et al. and Miranda et al studies (Aemaimanan et al., 2013; Miranda et al., 2017), the quantity of subgingival microbiota in DM patients was positively correlated with HbA1c. They stated that it is unknown whether hyperglycemia may directly alter the environmental in the pocket, causing colonization of certain pathogens, whether poor glycemic control stimulate hyper inflammation of in the pocket, favoring indirectly to colonization of those bacteria. Their finding is inconsistent with our result. The reason for this discrepancy could be related to different methodology (considering the quantity of bacteria in their studies and prevalence measurement in our study) and classification of the groups in their study. In Miranda et al study (Miranda et al., 2017), mean count of *Fn* and frequencies of detection of Tf was significantly higher in sites with probing depth ≥5 mm of the HbA1c ≥8% T2DM group compared with those of patients with HbA1c < 8%.

### 4.4 DM and periodontal parameters

In most of studies (Alasqah et al., 2018; Campus et al., 2005; Casarin et al., 2013), periodontal parameters (missing teeth, PD, PI, CAL, and BOP) in the diabetic group was significantly higher than in non‐diabetics. Which was consistent with the results of our study. The findings of our study and their studies confirm that periodontitis may be a chronic complication of DM and elevated glucose levels and glycation end products may alter the host response to bacterial infections.

One of the strengths of the present study is the comparison of the prevalence of pathogenic bacteria in different severity of periodontitis. Also, in this study, the effects that glycemic control and BMI level in diabetic patients may have on the subgingival microbiota have been considered and analyzed. Finally, it should be noted that one of the limitations of the present study was the lack of measurement of the concentration (mean count) of the studied bacteria. It is suggested that in future studies, in addition to frequency distribution, the concentration (mean count) of bacteria be measured and compared.

## 5 CONCLUSION

The results of the present study help to better understand the effects of DM on subgingival microbiota and also in relation to severity of periodontitis, glycemic control and BMI level. According to our result a higher frequency of detection of Aa was found in diabetic when compared to non‐diabetics. Glycemic control did not affect the frequency distribution of studied bacteria in T2DM. Pg was identified in higher frequency in overweight T2DM patients.

## CONFLICT OF INTEREST

The authors declare that there are no conflicts of interest in this study.

## AUTHOR CONTRIBUTIONS

Mojtaba Akherati, Hamid Abbaszadeh, and Ebrahim Shafaei have made substantial contributions to conception and design of the study. Mojtaba Akherati and Hamid Salehiniya have been involved in data collection and analysis. Hamid Abbaszadeh, Ebrahim Shafaei, Hamid Salehiniya and have been involved in data interpretation and drafting the manuscript. Mojtaba Akherati, Hamid Abbaszadeh, Ebrahim Shafaei and Hamid Salehiniya have critically revised the manuscript. All authors have given final approval of the version to be published.

## ETHICS STATEMENT

This study was approved by ethic committee of the University (ethic code: IR.BUMS.REC.1399.085). Study participants provided informed consent.

## Data Availability

The data that support the findings of this study are available from the corresponding author upon reasonable request.
